# Educational Interventions to Prepare Undergraduate Students for Out‐of‐Hours Practice as a Newly Qualified Doctor in the United Kingdom: A Scoping Review

**DOI:** 10.1111/tct.70299

**Published:** 2025-12-17

**Authors:** Alex Gordon, Molly Parkinson, Rosanna Watts, Kamal El‐Badawi, Erin Dawson, Rohan Chiktara, Lorna Burns, Nicola Brennan

**Affiliations:** ^1^ Torbay and South Devon NHS Foundation Trust, Torquay Devon UK; ^2^ Faculty of Health University of Plymouth Plymouth UK; ^3^ East Kent Hospitals University NHS Foundation Trust Kent UK; ^4^ Morriston Hospital Swansea UK

## Abstract

**Background:**

In the United Kingdom (UK), newly qualified doctors are routinely responsible for out‐of‐hours (OOH) care, often managing acutely unwell patients with limited supervision. Despite national recommendations encouraging OOH experience during medical school training, there is no formal curriculum for OOH practice.

**Objectives:**

This scoping review aimed to identify the range of educational interventions designed to prepare UK medical students for OOH clinical responsibilities and assess their reported effects on perceived and actual preparedness.

**Methods:**

Following Joanna Briggs Institute methodology, a systematic search of seven databases and grey literature sources was conducted. Eligible studies involved UK‐based medical students and reported empirical outcomes on educational interventions focused on OOH preparedness. Data were extracted and synthesised using the Template for Intervention Description and Replication checklist and Kirkpatrick's evaluation hierarchy.

**Results:**

Eighteen studies were included, primarily using quasi‐experimental designs of simulation‐based teaching. Interventions targeted a wide range of competencies, including prioritisation, communication, clinical decision‐making, and leadership. Thirteen studies demonstrated improvements in learner confidence or attitudes (Kirkpatrick Level 2a), while only three showed measurable performance improvement (Level 2b), and one demonstrated behavioural change (Level 3). Most lacked long‐term follow‐up. Grey literature analysis revealed inconsistent institutional expectations for OOH experience.

**Conclusions:**

Educational interventions on OOH are well‐received and enhance perceived preparedness. Current interventions often lack objective assessment and long‐term evaluation. This review was limited to published sources and may not reflect all current practice. Nationally standardised, longitudinal curricula supported by robust evaluation strategies are needed to improve graduate preparedness for OOH practice.

## 1 Introduction

Out‐of‐hours (OOH) work as a United Kingdom (UK)–based Postgraduate Year 1 or 2 doctor (referred to locally as foundation training) involves the direct care of patients on wards and emergency assessment units during evenings, overnight and at weekends. It is associated with responsibility for greater clinical complexity than regular working hours. It frequently involves exposure to the most unwell patients one will face during the early stages of postgraduate training, with the least amount of direct senior support from colleagues. Retrospective cohort studies at both single centre [1] and national multicentre levels [2] consistently show that emergency admission OOH is associated with an increased risk of mortality. Furthermore, a retrospective cohort study of almost 300,000 admissions to public hospitals across the whole of England demonstrated that there was an increased patient mortality associated with admission in the week following the first working day for new doctors [3].

This wider evidence highlights the intensity of OOH care. The periods when adverse outcomes are more likely coincide with the times when doctors at the very start of their careers are expected to contribute to patient care with the lowest levels of supervision. These shifts are typically characterised by reduced staffing, limited immediate access to senior input and a higher proportion of acutely unwell patients. For foundation doctors, this means encountering responsibilities that are qualitatively different from those in daytime work, requiring rapid assessment, prioritisation and autonomous decision‐making. Ensuring that new doctors are well prepared for these specific demands is therefore a central concern.

Preparedness for practice can be understood as a combination of knowledge, skills and behaviour that are required to meet a minimum standard of competence when newly qualified graduates start their professional careers [4]. A recent report into the preparedness of medical graduates identified gaps in abilities to undertake complex clinical decision‐making, communicate, manage uncertainty, lead and prioritise tasks [5]. In a qualitative study using an authentic on‐call simulation, Hawkins et al. (2021) [6] identified several determinants that hindered preparedness for the transition from student to doctor on‐call, including information overload, a reality gap between placements and practice and the challenges of working in unfamiliar environments, and argued that simulation could help mitigate these challenges.

Medical school curricula frequently do seek to address on‐call emergent scenarios through simulation‐based approaches. There is much literature and established practice that focuses on simulation to manage acutely unwell patients [7, 8], a common situation where foundation doctors may have enhanced responsibility out of hours. Similarly, there have been previous systematic reviews on isolated communication scenarios such as handover [9].

The concern is that, as predominant methods of preparing students for OOH, these may focus on individual skills or scenarios in isolation rather than considering the broader challenges of OOH working. In prior qualitative studies, medical students have commented that the areas they found challenging in OOH work include increased responsibility compared to in‐hours ward work, discrepancies between theoretical training and real‐world practice, the need for on‐the‐job learning and adaptation to unfamiliar environments [4, 6, 10]. Notably, these challenges are not framed as individual clinical tasks, but as the cumulative demands of the OOH setting—issues less amenable to short simulation exercises.

It may therefore be beneficial for educational interventions to not only expose students to address technical competencies in OOH contexts, but also take into account the aforementioned wider organisational and personal psychological factors to better support preparedness for on‐call duties. This is more in keeping with the perspective provided by Alexander et al. (2014) [11], who highlight that preparedness is influenced not only by individual competencies but also by broader workplace organisational factors and curriculum design.

In the UK, the General Medical Council (GMC—the UK medical regulator) recommends that students are encouraged to attend OOH work and that facilities are provided to allow this to happen. However, there is no formal recommendation about how this should be implemented [12]. There is also no current nationally defined syllabus for OOH work, nor is there a review of current practice to prepare UK medical graduates for this period of work.

*There is… no current nationally defined syllabus for OOH work*.

Given this lack of consensus, it is beneficial to compile current literature to describe what educational interventions are already established and their effectiveness in preparing medical students for practice in the on‐call setting through scoping review methodology.

The objectives of the review were:

RQ1. To describe the range of educational interventions available to prepare UK medical students for OOH work as a newly qualified doctor.

RQ2. To report the findings of any interventions on perceived and actual preparedness for practice.

## 2 Methods

### 2.1 Protocol and Registration

A review protocol was published in the public domain on 28 December 2022 and can be accessed via the Open Science Framework, accessible via https://osf.io/texaz/. The review was conducted in accordance with the Joanna Briggs Institute methodology for scoping reviews [13].

### 2.2 Eligibility Criteria

This question lends itself to a scoping review as the purpose is to scope a body of literature to establish what is known and identify knowledge gaps [14].

The elements of the review were structured using the Population, Concept, Context (PCC) framework (Table 1) [15].

**TABLE 1:** Inclusion and exclusion criteria for the review based on the Population, Concept, Context (PCC) framework.

	Inclusion criteria	Exclusion criteria
Population	Medical students enrolled on accredited UK medical degree programmes (any year of study).	Studies involving participants who had already graduated at the time of the intervention.
Concept	Educational interventions explicitly designed to improve knowledge, skills, attitudes or behaviours relevant to preparedness for out‐of‐hours (OOH) work. Eligible study designs included experimental, observational and qualitative approaches, provided they reported an educational intervention with outcomes in at least one of these domains.	Interventions that examined only feasibility, acceptability or cost, without evaluating impact on learners' knowledge, skills, attitudes or behaviours.
Context	Teaching on UK medical school programmes in which OOH refers to the practice environment of doctors in National Health Service hospitals outside of routine working hours. Interventions could take place in medical schools, teaching hospitals or associated education facilities.	Studies addressing skills outside the OOH setting or those targeting single tasks in isolation (e.g., handover training alone) without consideration of the broader demands of OOH work.
Other	Only peer‐reviewed full manuscripts published in English since 2009 were included.	Opinion pieces, non‐empirical reports or studies lacking outcomes in knowledge, skills, attitudes or behaviours were excluded

The inclusion of dates after 2009 represents the point at which the European Working Time Directive had been fully implemented in junior doctors' work schedules, dictating a significant change to the pattern of work towards shift work rather than prolonged periods of time in the workplace. It also represents the date of publication of the GMC's ‘Tomorrow's Doctors,’ which provided more centralised guidelines for the standards of education and training for medical students in the UK. While ‘Outcomes for Graduates’ has succeeded it in 2015 and 2018, ‘Tomorrow's Doctors’ represents a significant foundation that has influenced education policy since.

### 2.3 Information Sources

Embase, MEDLINE, CINAHL, ERIC, BEI, Scopus and Web of Science were searched on 15 April 2025. The full search strategy for MEDLINE is demonstrated in Appendix S1. We also evaluated grey literature from the GMC and Medical Schools Council websites. Forward and backward citation searching of included full texts was performed using Google Scholar.

### 2.4 Search

The search strategy for the healthcare databases comprised three blocks to represent the PCC framework. This included synonyms for medical students (P); OOH (C) and the UK (C) using a validated search filter [16]. The full search strategy is demonstrated in Appendix S1. This was constructed by one of the authors (L.B.), who is an information specialist.

### 2.5 Selection of Sources of Evidence

Screening and eligibility of titles and abstracts were undertaken blindly and independently by two authors (A.G. and M.P.) using Rayyan [17], with disagreements resolved by discussion. Full text review was conducted by two authors (A.G. and R.C.), with disagreements resolved by discussion with a third independent author (M.P.).

### 2.6 Data Charting Process

Data charting for specified outcomes and study characteristics was undertaken independently and in duplicate by A.G. and one of E.D., R.W., R.C. and K.E.‐B. using Google Forms, which were pre‐tested before use. Investigators of individual papers were not contacted for further information. Any discrepancies in data extracted between authors were checked against the manuscript by one author to determine accuracy.

While our protocol proposed tabulating and narratively synthesising findings by intervention type and outcome, we instead used the Template for Intervention Description and Replication (TIDieR) checklist and Kirkpatrick's evaluation hierarchy to provide a more structured and reproducible synthesis. This constituted a protocol deviation but enhanced consistency and comparability.

### 2.7 Data Items

The following data were extracted: participants, intervention location, study design, brief description of the intervention including stated aims and time course of evaluation. The skills, attitudes and behaviours assessed within each study were established, as well as a summary of study findings in the context of the research question.

The educational outcomes were graded according to Kirkpatrick's hierarchy. This is a framework used to evaluate the impact of the training intervention. The version of the framework used was that set out on the Best Evidence Medical Education Collaboration's specimen coding sheet [18].

As part of the grey literature review, responses from medical schools about their OOH training from Medical School Annual Returns were extracted from documents available on the GMC website.

### 2.8 Critical Appraisal of Individual Sources of Evidence

No critical appraisal of studies was performed as the purpose of the review was not to evaluate the efficacy of interventions but to describe the range of available interventions and their components [19].

### 2.9 Synthesis of Results

Extracted data were tabulated to describe individual studies. The framework for RQ1 was adapted from the TIDieR checklist [20]. Attitudes, skills and behaviours that each intervention purported to change and the Kirkpatrick hierarchy and findings for each study were tabulated for RQ2. Given the lack of consensus or national curriculum, we decided not to map this to any pre‐existing framework.

## 3 Results

### 3.1 Selection of Sources of Evidence

**FIGURE 1:**
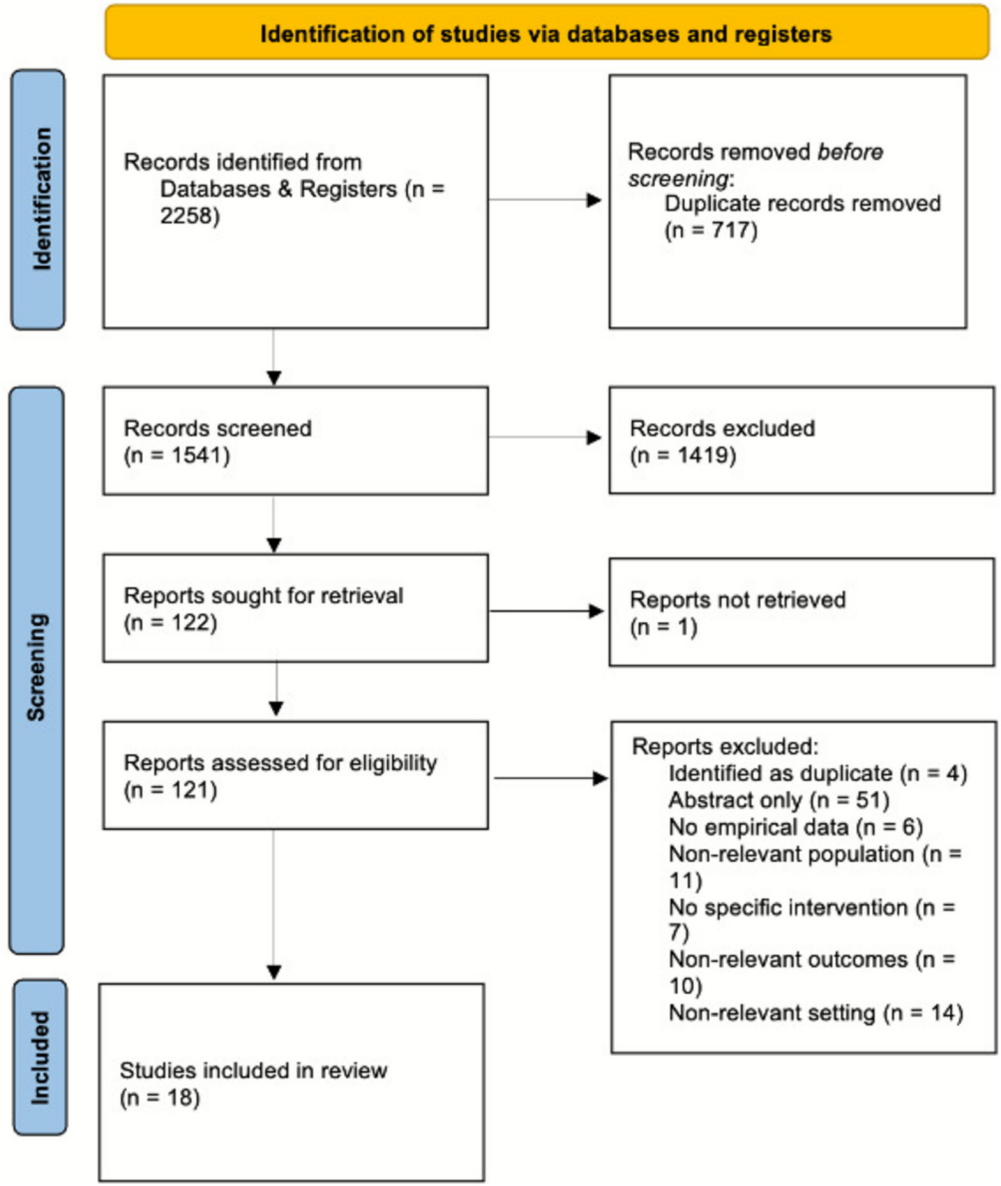
Study selection process presented using the PRISMA 2020 flow diagram. Figure adapted from Page et al. (2021) [21].

From 2258 records, 717 duplicates were removed, and 1541 records were screened. Following exclusions and retrieval, 121 reports were assessed for eligibility, resulting in 18 studies being included. The full study selection process is illustrated in the PRISMA flow diagram (Figure 1) [21].

#### 3.1.1 Study Characteristics

Table 2 summarises the 18 studies included in this review. The majority of studies (*n* = 15) employed a quasi‐experimental (before–after) design, while two used randomised controlled trials, and one was a post‐intervention qualitative evaluation with no pre‐assessment. The settings varied widely, encompassing hospitals, simulated hospitals, classrooms and virtual learning environments. Sample sizes ranged from 6 to 394 participants, with most studies focusing on final‐year medical students engaged in clinical placements or simulations of on‐call scenarios. Follow‐up intervals varied across the included studies, with the majority (14) reporting outcomes immediately post‐intervention, two studies assessing outcomes at 2 weeks and one study at 6 months.

**TABLE 2:** Description of included studies.

Authors	Population	Setting	Study design	Intervention description	Maximum follow‐up interval
Brown et al. (2016) [22].	Final‐year medical students (*n* = 30)	Hospital	Randomised trial	‘NightShift’ simulator (online simulation of a night shift)—participants had free use over a 2‐week study period. Developed from existing data on junior doctor activity.	2 weeks
Darbyshire et al. (2013) [23]	Senior medical students (*n* = NS)	Classroom	Quasi‐experimental (before–after)	1‐h session on handover at the start of an out‐of‐hours shift	Immediate
Desouky et al. (2024) [24]	Final‐year medical student (*n* = 72)	Hospital	Quasi‐experimental (before–after)	9‐station simulated programme with bleep system	Immediate
Dickinson et al. (2014) [25]	Final‐year medical students (*n* = NS)	Hospital	Quasi‐experimental (before–after)	Pager simulation for 4 h—students given bleep and expected to respond to messages while being on normal clinical placement	Immediate
Emin et al. (2022) [26]	Medical students in clinical training (*n* = 41)	Virtual Learning Environment	Quasi‐experimental (before–after)	Virtual on‐call. Undertake on‐call tasks in groups who are given handover sheet consisting of five scenarios with two groups observing	Immediate
Fisher et al. (2014) [27]	3rd‐year medical students (*n* = 56)	Hospital	Quasi‐experimental (before–after)	Teaching session: 10 students per session Prior learning material on telephone comms and SBAR Each student had scenario to respond to via pager	Immediate
Hamilton et al. (2021) [28]	Final‐year medical students (*n* = NS)	Simulated hospital	Quasi‐experimental (before–after)	Simulation setting, role playing one doctor and one pharmacist and three nurses for three wards. Participants undertook mock scenarios, lasting 1 h each. Followed by debrief and questionnaire	6 months
Harrison et al. (2021) [29]	Final‐year medical students (*n* = 25)	Virtual learning environment	Quasi‐experimental (before–after)	Delivered by junior doctors Initial introductory presentation 2:1 participant to facilitator ratio in breakout room where multiple bleeps for patient review simulated Debrief	Immediate
Manalayil et al. (2020) [30]	Final‐year medical students (*n* = 17)	Hospital	Quasi‐experimental (before–after)	Simulation of multiple on‐call tasks	Immediate
Misquita et al. (2020) [31]	Final‐year medical students (*n* = 38)	Hospital	Quasi‐experimental (before–after)	Students went to real wards, reviewed simulated patients' notes and formulated and documented management plans Two simulation sessions 2 weeks apart where students given a paging device and go round hospital; one acute scenario, gather information to assess urgency of task via bleep and prioritise.	2 weeks
McGlynn et al. (2012) [32]	Medical students (*n* = NS)	Simulated hospital	Qualitative evaluation	Students do 45‐min simulated on‐call following handover	
Moqeem et al. (2023) [33]	Final‐year medical students (*n* = 20)	Hospital	Quasi‐experimental (before–after)	6‐month shadowing programme	Immediate
Nichols et al. (2024) [34]	4th‐ and 5th‐year medical students (*n* = 35)	Hospital	Quasi‐experimental (before–after)	Three simulation sessions with five clinical scenarios each	Immediate
Nitiahpapand et al. (2020) [35]	Final‐year medical students (*n* = 20)	Hospital	Quasi‐experimental (before–after)	Six‐station high‐fidelity simulated virtual on‐call; two low‐priority scenarios in rotation	
Ramsden et al. (2016) [36]	Final‐year medical students (*n* = 34)	Hospital	Quasi‐experimental (before–after)	An hour on‐call—during the hour, they received a handover, carried out outstanding ward tasks, responded to bleeps and assessed an acutely unwell patient, before handing patient care onto colleagues. Following the simulation, there was time for discussion and feedback.	Immediate)
Seale et al. (2019) [37]	Final‐year medical students (*n* = 50)	Classroom	Randomised trial	1‐h teaching session on telephone communication skills and the prioritisation of job lists 10‐min simulation session	Immediate
Scott et al. (2024) [38]	Clinical‐year medical students (*n* = 394)	Hospital	Quasi‐experimental (before–after)	2‐h session comprising a compilation of 10 of 27 possible scenarios. 30‐min debrief	Immediate
Tano and Laraman (2022) [39]	Final‐year medical students (*n* = 6)	Hospital	Quasi‐experimental (before–after)	1 h, went to ward, carry out written tasks including management plan, interpreting data, prescribing and escalating concerns as necessary to facilitators. Session ends with mock handover and debrief	Immediate

#### 3.1.2 RQ1: Educational Intervention Components

The majority of interventions (*n* = 17) utilised simulation as the primary teaching method, with only two studies incorporating stand‐alone teaching sessions or combining didactic input with simulation [22, 37] (Table 3). Most interventions were developed and delivered by medical school faculty, NHS education staff or clinical tutors, although several were led by junior doctors as near‐peer facilitators [29, 30], and one was delivered by an international faculty team [26].

**TABLE 3:** Components of educational interventions in studies and findings from evaluation.

	Setting	Modality and fidelity	Format and content	Facilitators	Debriefing	Assessment method	Findings	Highest level on Kirkpatrick hierarchy^a^
Brown et al. (2016) [22].	Nottingham City Hospital (large teaching hospital)	Virtual simulation, tablet‐based, lower fidelity (serious video game)	Simulated night shift using real task data (> 100 k tasks); included task prioritisation, navigation, interruptions, fatigue element; free access during induction	Research team developed, delivered to final‐year students	Minimal; focus group and feedback post‐use	Task data via NerveCentre (time to complete urgent/non‐urgent tasks); focus group + online feedback	Intervention group completed non‐urgent tasks faster than control (85 vs. 158 min, *p* = 0.027); no effect on urgent tasks; users reported realism but also frustration with navigation and fatigue features	3
Darbyshire et al. (2013) [23]	Classroom, small group (6–8 students typical)	Role play, discussion, video	1‐h session: introduction, group discussion of experiences; role‐plays of handover in pairs; video of good vs. poor handover; multi‐disciplinary role play; reflection task (attend handover)	University tutors	Peer + facilitator feedback during role plays; group reflections	Post‐session Likert feedback (clear objectives, relevance, interactivity); self‐reported knowledge gain	High satisfaction (mean scores ~9/10 for relevance, clarity, interactivity); students reported improved knowledge of handover (mean 9.1/10); positive qualitative feedback	2a
Desouky et al. (2024) [24]	Royal Blackburn Hospital, UK—rotating stations over 5 days	High‐fidelity immersive sim (nine interactive stations, including bleep interruptions)	Students rotate through nine on‐call stations (e.g., managing unwell patients, DKA prescribing, DNACPR discussions), testing prioritisation and decision‐making	Clinical simulation facilitators and educators	Not explicitly stated	Pre‐ and post‐event self‐reported confidence and preparedness via 25‐item Likert questionnaire	Significant improvements across all domains; largest effect sizes in task prioritisation (*r*_s_ = 0.74), managing unwell patients (*r*_s_ = 0.60) and escalation skills (*r*_s_ = 0.49)	2a
Dickinson et al. (2014) [25]	Royal Preston Hospital, Lancashire Simulation Centre	Simulation, high‐fidelity ward environment with pagers	Final‐year students allocated pager for 4 h during placement; respond to random pages (lab results, cannula, referrals, simulated patient scenarios); culminates in group cardiac arrest scenario	Simulation centre staff, clinical education team	Group debrief after arrest scenario, based on written logs; peer + faculty feedback	Observation logs of responses, telephone manner, prioritisation, management decisions; qualitative feedback	Students valued realism and unpredictability; reported it felt like being an FY1 (‘flying solo’); feedback highlighted importance of prioritisation, delegation, communication	1
Emin et al. (2022) [26]	Multicentre (UK, Greece, Turkey, India)	Virtual hospital simulation, high‐fidelity	Three cycles of scenarios (handover sheets: two non‐urgent, three urgent tasks); students rotated performing/observing; tasks included prioritisation, communication, escalation	International faculty team	Structured peer + faculty‐led debrief	TEAM tool (validated team performance scale)	Global TEAM scores improved across cycles; handover performance weakest; statistical significance limited by sample size	2b
Fisher et al. (2014) [27]	North Tyneside General Hospital, UK	Simulation with low‐cost props (pager, speakerphone, role‐play nurse; one high‐fidelity resuscitation scenario)	Students paged by role‐played nurse, responded via speakerphone (audible to group); scenarios on acutely unwell patients with infections; final task: telephone handover to registrar	Clinical tutors + nurse practitioners	Student‐led debrief with tutor facilitation; Pendleton's model + advocacy/enquiry approach	Pre/post confidence ratings (10‐point Likert); feedback questionnaire; free‐text comments	Significant increases in confidence for telephone interaction (median 4 → 7, *p* < 0.001) and handover (5 → 7, *p* < 0.001); feedback valued realism and opportunity to practise	2a
Hamilton et al. (2021) [28]	NHS Lanarkshire; ward‐based	High‐fidelity interprofessional ward simulation	1‐h multidisciplinary on‐call scenarios; students from medicine, nursing, pharmacy; tasks: prioritisation, managing deteriorating patients, communication	NHS medical education staff	Profession‐specific debriefs followed by multidisciplinary group debrief	Immediate post‐session +6‐month follow‐up questionnaires	Immediate positive feedback (confidence, prioritisation, teamwork, identity); partial retention of gains at 6 months, but technical skills declined.	2a
Harrison et al. (2021) [29]	University of Liverpool, online	Virtual simulation via Zoom	2‐h ‘virtual on‐call’: intro presentation → breakout rooms (2:1 student: facilitator); simulated bleeps requiring prioritisation; concluding group presentation	Junior doctors (near‐peer facilitators)	Participant‐centred debrief within breakout rooms (Pendleton model)	Pre/post self‐reported confidence (Wilcoxon signed‐rank test)	Significant improvements in confidence for all scenarios (*p* < 0.01); pre‐course 75% unconfident vs. post‐course 4%; 100% would recommend	2a
Manalayil et al. (2020) [30]	Blackpool Victoria Hospital, UK	Ward‐based low–medium fidelity simulation (students carried hospital bleep for 1 h)	Students carried a pager, received multiple simulated ‘nurse’ calls with too many tasks to complete in time; tasks included prescribing, reviewing results, discharge summaries, confirming death and managing an acutely unwell patient	Junior doctors and faculty	Facilitated group debrief using Gibb's reflective cycle; discussion of coping strategies, prioritisation and communication	Post‐session Likert questionnaire + free‐text comments	100% reported increased confidence; valued realism (open wards, unfamiliar environment); strongly recommended programme expansion	2a
Misquita et al. (2020) [31]	Chelsea and Westminster Hospital, London, UK	High‐fidelity ward‐based simulation with bleeps	Two 3‐h sessions, 2 weeks apart. Students given a pager, independently managed simulated calls from nurses (role‐played). Tasks included prescribing, interpreting results, IV fluids, AKI, MI, sepsis, PE. One acute scenario per session.	Clinical facilitators (1:1 ratio)	One‐to‐one personalised debriefing, written feedback, handouts	Pre/post confidence questionnaires (Likert), performance assessment of telephone handover (SBAR) and acute patient assessment (ABCDE), thematic analysis of focus groups	↑ Confidence and preparedness in all six skills (*p* < 0.001), ↑ performance in handover and acute assessment (*p* < 0.001). Improvements sustained at 2 months. Students valued independence, realism, responsibility. Programme seen as more useful than seminars/shadowing.	2b
McGlynn et al. (2012) [32]	NHS Lanarkshire, Scotland, UK	High‐fidelity ward‐based simulation with bleeps	Individual 45‐min ‘evening on‐call’ session: students received handover list + pager, managed tasks (e.g., sedation prescribing, ECG/blood interpretation, GI bleed) across mock wards	Experienced clinicians + clinical skills facilitators	Immediate structured individual feedback from observer (doctor) using pre‐developed template	Student evaluation questionnaires (Likert), free‐text comments; feedback from observers	Students reported session among the best ways to learn; felt better prepared for FY1 role; valued realism, responsibility and individual feedback	2a
Moqeem et al. (2023) [33]	Royal Surrey NHS Foundation Trust, UK	Real‐life twilight shadowing (clinical placement)	Fortnightly twilight shifts over 6 months shadowing an F1 on‐call; focus on handover, hospital at night coordination, crash team exposure	Undergraduate education committee	Not specified	Pre/post self‐reported confidence via 1–10 scale; feedback survey	Confidence increased from 3.2 to 6.95 (non‐significant, *p* = 0.15); 100% would recommend; valued realistic hands‐on exposure	2a
Nichols et al. (2024) [34]	Two hospital sites in UK	Low‐fidelity, ward‐based simulation	Three sessions each with five written on‐call tasks (medical/surgical); bleeps; student‐led handover after tasks	30 resident (junior) doctors	Structured facilitator‐led debrief	Pre/post questionnaires (quantitative + qualitative)	Statistically significant improvements in confidence across all domains (*p* < 0.05); constructive learning environment; multiple sessions enhanced confidence further	2a
Nitiahpapand et al. (2020) [35]	Bedford Hospital Trust, UK	High‐fidelity, six‐station virtual on‐call with pager interruptions	Six core stations: emergency collapse, warfarin prescribing, chest X‐ray interpretation, IV fluids, test requests, prescribing; plus low‐priority distractor tasks (e.g., discharge letter)	Clinical education team	Not explicitly reported	Pre/post Likert questionnaire across 10 domains; paired *t* test; Cronbach's *α*	Significant ↑ in 8/10 domains (confidence, knowledge, situational awareness, decision making, communication, leadership, stress management, coping with fatigue); no change in technical skills/teamwork; reliable questionnaire (*α* = 0.8+)	2a
Ramsden et al. (2016) [36]	The Lister Hospital, Stevenage, UK	Ward‐based, high‐fidelity simulation	2‐h programme: briefing → 1‐h simulation where students acted as FY1 on‐call (handover, ward tasks, bleeps, assess unwell patient, handover again) → discussion and feedback	Two doctors per session	Group discussion and feedback	Pre/post questionnaires on preparedness and confidence	94% felt more confident post‐course; 100% recommended inclusion in curricula; students valued realism, improved time management, handover, prioritisation and acute care skills	2a
Seale et al. (2019) [37]	GKT School of Medical Education, King's College London, UK	Ward‐based simulation with telephone handovers (low–medium fidelity)	Group A received structured teaching on telephone skills + prioritisation; Group B received none. All students then undertook an on‐call simulation with five tasks, bleeps, distractions and final emergency call	Clinical skills tutors	Not explicitly detailed (structured observation and feedback implied)	Objective observation checklists (info asked/recorded, prioritisation accuracy); post‐simulation Likert questionnaire	Trained group significantly outperformed untrained in info gathering and prioritisation (*p* < 0.001); higher confidence in call‐handling; all students valued inclusion in curriculum	2b
Scott et al. (2024) [38]	Eight UK hospitals (multi‐site)	High‐fidelity, ward‐based	Circuit of written on‐call scenarios with ‘bleeped’ tasks (emergencies, prescribing, distractions); students could escalate by phone	Local faculty at each hospital site	30‐min structured debrief on prioritisation and communication	Pre/post Likert rating of preparedness; subset of 20 paired ratings for specific skills	Student preparedness improved significantly (pre 4/10 → post 7/10, *p* < 0.01); skill‐specific confidence increased	2a
Tano and Laraman (2022) [39]	Aneurin Bevan University Health Board, Wales, UK	Ward‐based simulation (medium fidelity, 1 h)	Six final‐year students: briefing → device handover → multiple ward tasks (management plan, interpreting data, prescribing, escalation) + distractor tasks → mock handover	Clinical education team	Group debrief after mock handover	Pre/post self‐rated questionnaires (confidence, preparedness, SBAR, prescribing, decision‐making, etc.)	↑ Confidence and preparedness (confidence +58%, preparedness +42%); gains across decision‐making, prioritisation, SBAR, prescribing	2a

Abbreviations: ABCDE, airway, breathing, circulation, disability, exposure; AKI, acute kidney injury; DKA, diabetic ketoacidosis; DNACPR, do not attempt cardiopulmonary resuscitation; ECG, electrocardiogram; EWS, Early Warning Score; F1, Foundation Year 1 doctor; FY1, Foundation Year 1 doctor; GI, gastrointestinal; IV, intravenous; iVOC, international virtual on‐call; MI, myocardial infarction; NHS, National Health Service; NOS, not otherwise specified; OOH, out‐of‐hours; PE, pulmonary embolism; SBAR, Situation, Background, Assessment, Recommendation; Sim, simulation; TEAM, Team Emergency Assessment Measure; UK, United Kingdom; VOC, virtual on‐call.

^a^ Kirkpatrick's Hierarchy of Educational Outcomes: Level 1—Reaction: learners' immediate response or satisfaction; Level 2a—Learning (attitudes/confidence/preparedness): self‐reported changes in knowledge, skills or attitudes; Level 2b—Learning (knowledge/skills): objective demonstration of knowledge or skill gain via structured or validated assessment; Level 3—Behaviour: transfer of learning into behaviour within a practice or simulated environment; Level 4—Results: impact on organisational practice or patient outcomes (not reported in included studies).

*The majority of interventions (n = 17) utilised simulation as the primary teaching method*.

Educational theories underpinning the interventions were inconsistently reported. Six studies referenced specific frameworks, including Gagné's nine events of instruction [23], Kolb's experiential learning theory [30], situated learning [25], Pendleton's feedback guidelines [27, 29] and the Johari Window model [34]. The remaining studies did not cite any explicit theoretical basis to their session structure or feedback. Learning objectives were reported in eight studies [23, 24, 25, 27, 28, 30, 31, 36] and frequently focused on clinical prioritisation, decision‐making, communication, teamwork and patient safety in the context of OOH care.

Session structures ranged in complexity and duration. Most interventions featured a mix of simulated clinical scenarios, communication tasks, paging systems and structured debriefing. Some incorporated additional elements such as didactic teaching [23, 37], peer reflection [34], role play [23] or repeated sessions across time [31, 33, 34], while one study employed real‐world shadowing of Foundation Year 1 doctors during twilight shifts [33].

Scenarios that formed part of the interventions commonly addressed tasks such as managing unwell patients, prescribing, handover, responding to bleeps and interdisciplinary communication. Several designs deliberately introduced time pressure, multiple simultaneous tasks or distractors to simulate the intensity of OOH shifts [30, 35, 37, 38, 39].

Outcomes were categorised using the Kirkpatrick hierarchy [18]. A single study reported Level 1 outcome (reaction of students to the intervention) [25]. Thirteen studies reported Level 2a outcomes (self‐reported confidence, attitudes or preparedness) [23, 24, 27, 28, 29, 30, 32, 33, 34, 35, 36, 38, 39]. Three studies reached Level 2b by incorporating structured or validated performance measures (e.g., TEAM tool, observed handover accuracy, structured acute assessment) [26, 31, 37]. One study reported a Level 3 outcome, demonstrating behavioural impact within a simulated environment through increased efficiency in completing ward tasks [22].

Overall, student feedback was consistently positive. Participants described the interventions as realistic [25, 32, 36, 38, 39] and directly relevant to their future clinical roles. Many valued the opportunity to practise decision‐making and prioritisation [24, 30, 34, 35, 37], as well as the time management in a simulated OOH context [30, 36, 39]. Across studies, the most frequently reported benefit was improved confidence and a stronger sense of preparedness for independent practice as a Foundation Year 1 doctor [22, 29, 32, 33].

#### 3.1.3 RQ1: Grey Literature Responses

Verbatim responses from Medical School Annual Returns to the GMC from 2017/2018 were available in the public domain (Appendix S2). Within this, medical schools were required to specifically comment on their approach to OOH via free text. The responses indicate a broad range of approaches to provision, with variability between institutions regarding the extent to which OOH is compulsory. These can generally be categorised as per Table 4.

**TABLE 4:** Medical school mandates for OOH attendance.

OOH attendance requirement	Medical schools
Compulsory/requirement (14)	Plymouth, Sheffield, Birmingham, Liverpool, Cambridge, Newcastle, Aberdeen, Southampton, St. George's, Buckingham, Glasgow, Leeds, Keele, Oxford
Specific rota'ing in (3)	Warwick, Imperial, Leicester
Expectation of attendance only (2)	Barts, Cardiff
Offer of attendance (5)	Lancaster, University of East Anglia, Swansea, Manchester, Edinburgh
Not specified (2)	King's College London, University College London

Abbreviation: OOH, out‐of‐hours.

#### 3.1.4 RQ2: The Skills/Knowledge, Attitudes and Behaviours Measured in Each Study

The studies collectively assessed a broad range of clinical and professional proficiencies relevant to OOH preparedness (Box 1). Commonly measured skills included clinical decision‐making, task prioritisation, management of acutely unwell patients, telephone communication, prescribing, documentation, data interpretation and the use of bleeps. Technical skills such as acute assessment and intervention were measured alongside a wide array of non‐technical skills, including teamwork, leadership, adaptability, situational awareness and information gathering.

Box 1 **List of proficiencies (attitudes/skills/behaviours) directly measured in studies.**

Time taken to complete urgent tasks [22].

Time taken to complete non‐urgent tasks [22].

Handover of care [23, 24, 34, 38].

Acutely unwell patient management [24, 25, 34].

Clinical decision‐making/clinical management [24, 25, 28, 29, 31, 35, 39].

Prioritisation [24, 25, 26, 28, 30, 34, 37, 38, 39].

Phone communication [25, 27, 31, 37].

Bleep use [38].

Speed of response to page [25].

Time management [28].

Communication [24, 28, 30, 32, 35].

Gathering information [38].

Prescribing [28, 34].

Documentation [28].

Situational awareness [35].

Stress management [35].

Coping with fatigue [35].

Working under pressure [39].

Data interpretation [24, 39].

Recognising limitations [28].

Teamwork [26, 27, 30, 35].

Adaptability [26].

Leadership [26, 35].

In terms of attitudes, many studies focused on students' self‐perceived confidence, preparedness and awareness of their future responsibilities as junior doctors. Reported behaviours included the ability to work under pressure, manage time effectively, respond to bleeps promptly, escalate concerns appropriately and cope with stress and fatigue during high‐demand scenarios.

## 4 Discussion

This scoping review explored the range and effectiveness of educational interventions designed to prepare UK medical students for OOH work as foundation doctors.

We identified that short‐term, often single‐session, simulation‐based methods are predominantly used to address a mix of skills, attitudes and behaviours that are perceived as learning needs by students, faculty and wider literature on under preparedness in UK medical graduates [4, 10]. This consistency suggests that the educational drivers for developing such interventions are aligned at both local and national levels and supported by existing scholarship.

*We identified that short‐term, often single‐session, simulation‐based methods are predominantly used*.

Despite the widely appreciated need for education in this area, most of the assessments of the effectiveness of these interventions are based on immediate post‐session student perception of acquired competence. Most reported outcomes were situated at Kirkpatrick Level 2a (confidence and self‐perception). Student perceptions of their own competences, particularly if they are inexperienced or poorly performing, are known to poorly correlate with more objective measures of preparedness [40]. This limits insight into actual proficiency gained through the identified teaching methods, especially given the lack of long‐term follow‐up we have also identified. As such, current evidence offers little clarity on how well these interventions prepare students for real on‐call duties.

*Most of the assessment of the effectiveness of these interventions is based on immediate post‐session student perception*.

A further limitation of the short‐term, simulation‐based approaches that dominate the literature is that they overlook the broader view of preparedness as an outcome of individual competence, curricula factors and organisational factors described by Alexander et al. (2014) [11]. Most interventions concentrated on situational proficiency within brief 1‐ to 2‐h sessions but gave little attention to the sustained pressures that impair performance during real OOH work. Important human factors, such as managing sleep deprivation [41, 42] and decision fatigue [43], were not addressed by any studies in our review. These are vital to effective OOH performance and depend on a complex interplay of personal, social and systemic factors—dimensions that extend beyond what isolated simulations typically capture.

Even if higher levels of Kirkpatrick achievement were reported, international literature suggests that the proficiencies most frequently assessed do not fully capture what it means to be prepared for on‐call work. For example, recently qualified Australian graduates highlight that they struggle with seeking support and felt that there was an overemphasis on theoretical training at detraction from the practicalities of the clinical environment, rather than identifying deficiencies in discrete technical skills [44]. A Dutch study also found that prolonged exposure to clinical practice prior to starting out of hours improved feelings of preparedness, rather than simulation‐based approaches that provide only a subset of technical skills [45]. These perspectives further highlight that preparedness for OOH work extends beyond the technical skills emphasised in most simulation‐based interventions, requiring attention to broader experiential, organisational and relational factors.

### 4.1 Implications for Research and Practice

This review highlights the need for more rigorous evaluation of OOH interventions. Future studies should move beyond Kirkpatrick Level 2a outcomes, incorporating objective performance assessments, validated tools and longitudinal follow‐up to better capture skill retention and behavioural change. Approaches such as structured workplace‐based assessments or multi‐source feedback could provide more meaningful insights into preparedness for real on‐call duties.

Curricular design would also benefit from greater theoretical grounding. Using established frameworks such as experiential learning or instructional design models could improve reproducibility, transparency and comparability across institutions. Given the neglected role of human factors, resilience and decision‐making under sustained pressure, future interventions should broaden their scope beyond short, isolated simulations to more authentically reflect the demands of OOH practice.

At the organisational and policy level, the lack of national guidance and inter‐medical school variability in OOH exposure remain barriers to widespread improvements in graduate preparedness for OOH work. Establishing minimum standards for OOH exposure within UK medical schools could help reduce institutional variability and ensure equity in graduate preparedness, while still allowing local adaptation and innovation. Similar initiatives in other health systems facing comparable transition challenges could also provide useful lessons.

*The lack of national guidance and inter‐medical school variability in OOH exposure remain barriers to widespread improvements*.

### 4.2 Limitations

A scoping review, as a methodological approach, is valuable for identifying the breadth of existing knowledge within a particular area. In this review, we identified that simulation‐based approaches to OOH preparation are common, but that most reported outcomes were short‐term and limited to learner perceptions.

By design, however, a scoping review does not determine the effectiveness of specific interventions or identify optimal methods for enhancing competence. Nor does it provide the depth of qualitative synthesis that might be required to fully explore why certain approaches are perceived as more effective or meaningful to learners.

This review also relied on publicly available literature and documents, which may not fully capture current practice across all UK medical schools. Unpublished interventions or local innovations or those captured only in conference proceedings are likely to exist but are not represented in the peer‐reviewed literature. Developments in curricula since the 2017/2018 GMC returns may similarly be under‐reported. As such, the evidence summarised here reflects only what is available in the public domain rather than the totality of educational practice.

## 5 Conclusions

Simulation of the on‐call setting is an enjoyable and valuable learning experience for medical students. However, current evidence is limited by a reliance on short‐term self‐reported outcomes and a lack of longitudinal follow‐up, meaning little is known about the sustained impact of these programmes on skills, attitudes or behaviours in real on‐call practice. Future research should incorporate objective measures and longer‐term follow‐up to clarify effectiveness, while curricular design should broaden beyond technical proficiency to include human factors and the sustained pressures of OOH work. At the policy level, clearer national guidance and more consistent expectations for OOH exposure across UK medical schools could help ensure equitable preparation for foundation training.

*Current evidence is limited by a reliance on short‐term self‐reported outcomes and a lack of longitudinal follow‐up*.

## Author Contributions

**Alex Gordon:** conceptualisation, investigation, formal analysis, methodology, writing – original draft, writing – review and editing. **Molly Parkinson:** conceptualisation, investigation. **Rosanna Watts:** investigation, writing – review and editing. **Kamal El‐Badawi:** investigation. **Erin Dawson:** investigation. **Rohan Chiktara:** investigation. **Lorna Burns:** conceptualisation, methodology, writing – review and editing, supervision. **Nicola Brennan:** conceptualisation, methodology, writing – review and editing, supervision.

## Funding

The authors have nothing to report.

## Conflicts of Interest

The authors declare no conflicts of interest.

## Supporting information


**Appendix S1:** Search Strategy for Medline

**Appendix S2:** Medical School Annual Return Responses 2017/2018

## Data Availability

Data sharing not applicable to this article as no datasets were generated or analysed during the current study.

## References

[1] F. Maggs and M. Mallet , “Mortality in Out‐of‐Hours Emergency Medical Admissions—More Than Just a Weekend Effect,” Journal of the Royal College of Physicians of Edinburgh 40 (2010): 115–118, 10.4997/jrcpe.2010.205. 21125051

[2] P. Aylin , A. Yunus , A. Bottle , A. Majeed , and D. Bell , “Weekend Mortality for Emergency Admissions. A Large, Multicentre Study,” Quality & Safety in Health Care 19 (2010): 213–217, 10.1136/qshc.2008.028639. 20110288

[3] M. H. Jen , A. Bottle , A. Majeed , D. Bell , and P. Aylin , “Early In‐Hospital Mortality Following Trainee Doctors' First Day at Work,” PLoS ONE 4 (2009): e7103, 10.1371/journal.pone.0007103. 19774078 PMC2743809

[4] L. V. Monrouxe , L. Grundy , M. Mann , et al., “How Prepared Are UK Medical Graduates for Practice? A Rapid Review of the Literature 2009–2014,” BMJ Open 7 (2017): e013656, 10.1136/bmjopen-2016-013656. PMC5253586 28087554

[5] T. Gale , N. Brennan , and N. Langdon , Preparedness Of Recent Medical Graduates to Meet Anticipated Healthcare Needs (General Medical Council, 2022), https://www.gmc‐uk.org/‐/media/documents/p4p‐research‐final‐report‐feb22_pdf‐89855094.pdf.

[6] N. Hawkins , H.‐C. Younan , M. Fyfe , R. Parekh , and A. McKeown , “Exploring Why Medical Students Still Feel Underprepared for Clinical Practice: A Qualitative Analysis of An Authentic On‐Call Simulation,” BMC Medical Education 21 (2021): 165, 10.1186/s12909-021-02605-y. 33731104 PMC7972243

[7] I. Motola , L. A. Devine , H. S. Chung , J. E. Sullivan , and S. B. Issenberg , “Simulation in Healthcare Education: A Best Evidence Practical Guide. AMEE Guide No. 82,” Medical Teacher 35 (2013): e1511–e1530. 20130813, 10.3109/0142159x.2013.818632. 23941678

[8] H. R. Church , D. Murdoch‐Eaton , and J. Sandars , “Under‐ and Post‐Graduate Training to Manage the Acutely Unwell Patient: A Scoping Review,” BMC Medical Education 23 (2023): 146, 10.1186/s12909-023-04119-1. 36869334 PMC9983517

[9] M. Gordon , E. Hill , J. N. Stojan , and M. Daniel , “Educational Interventions to Improve Handover in Health Care: An Updated Systematic Review,” Academic Medicine 93 (2018): 1234–1244, 10.1097/ACM.0000000000002236. 29620675 PMC6092095

[10] J. C. Illing , G. M. Morrow , C. R. Rothwell nee Kergon , et al., “Perceptions of UK Medical Graduates' Preparedness for Practice: A Multi‐Centre Qualitative Study Reflecting the Importance of Learning on the Job,” BMC Medical Education 13 (2013): 34, 10.1186/1472-6920-13-34. 23446055 PMC3599362

[11] C. Alexander , J. Millar , N. Szmidt , K. Hanlon , and J. Cleland , “Can New Doctors Be Prepared for Practice? A Review,” Clinical Teacher 11 (2014): 188–192, 10.1111/tct.12127. 24802919

[12] General Medical Council . “Student Assistantships. Guidance on Undergraduate Clinical Placements,” gmc‐uk.org, (2022), pp. 32–33.

[13] M. D. J. Peters , C. Marnie , A. C. Tricco , et al., “Updated Methodological Guidance for the Conduct of Scoping Reviews,” JBI Evidence Synthesis 18 (2020): 2119–2126, 10.11124/jbies-20-00167. 33038124

[14] Z. Munn , D. Pollock , H. Khalil , et al., “What Are Scoping Reviews? Providing a Formal Definition of Scoping Reviews as a Type of Evidence Synthesis,” JBI Evidence Synthesis 20, no. 4 (2022): 950–952, 10.11124/jbies-21-00483. 35249995

[15] M. D. J. Peters , C. Godfrey , P. McInerney , Z. Munn , A. C. Tricco , and H. Khalil , “Scoping Reviews (2020),” in JBI Manual for Evidence Synthesis, ed. E. Aromataris , C. Lockwood , K. Porritt , B. Pilla and Z. Jordan (JBI, 2024), https://synthesismanual.jbi.global, 10.46658/JBIMES-24-09.

[16] L. Ayiku , P. Levay , T. Hudson , et al., “The Embase UK Filter: Validation of a Geographic Search Filter to Retrieve Research About the UK from OVID Embase,” Health Information and Libraries Journal 36, no. 2 (2019): 121–133, 10.1111/hir.12252. 30912233

[17] M. Ouzzani , Z. Hammady , Z. Fedorowicz , and A. Elmagarmid , “Rayyan—A Web and Mobile App for Systematic Reviews,” Systematic Reviews 5 (2016): 210, 10.1186/s13643-016-0384-4. 27919275 PMC5139140

[18] S. Yardley and T. Dornan , “Kirkpatrick's Levels and Education ‘Evidence’,” Medical Education 46 (2012): 97–106, 10.1111/j.1365-2923.2011.04076.x. 22150201

[19] Z. Munn , M. D. J. Peters , C. Stern , C. Tufanaru , A. McArthur , and E. Aromataris , “Systematic Review or Scoping review? Guidance for Authors When Choosing Between a Systematic or Scoping Review Approach,” BMC Medical Research Methodology 18 (2018): 143, 10.1186/s12874-018-0611-x. 30453902 PMC6245623

[20] T. C. Hoffmann , P. P. Glasziou , I. Boutron , et al., “Better Reporting of Interventions: Template for Intervention Description and Replication (TIDieR) Checklist and Guide,” BMJ (Clinical Research Ed.) 348 (2014): g1687, 10.1136/bmj.g1687. 24609605

[21] M. J. Page , J. E. McKenzie , P. M. Bossuyt , et al., “The PRISMA 2020 Statement: An Updated Guideline for Reporting Systematic Reviews,” BMJ (Clinical Research Ed.) 372 (2021): n71. 20210329, 10.1136/bmj.n71. PMC8005924 33782057

[22] M. Brown , J. Pinchin , R. Valand , et al., “NightShift Simulation to Train Newly Qualified Doctors in Non‐Technical Skills: A Feasibility Study,” Future Hospital Journal 3 (2016): 94–98, 10.7861/futurehosp.3-2-94. 31098195 PMC6465818

[23] D. Darbyshire , M. Gordon , and P. Baker , “Teaching Handover of Care to Medical Students,” Clinical Teacher 10, no. 1 (2013): 32–37, 10.1111/j.1743-498X.2012.00610.x. 23294741

[24] O. Desouky , N. Lawes , T. Hunter , N. Zafar , and W. Whitehead , “On‐Call Simulation: A One‐Day Comprehensive Simulation of Clinical Practice for Final‐Year Medical Students,” Cureus 16 (2024): e74555. 39735066 10.7759/cureus.74555 PMC11672163

[25] M. Dickinson , M. Pimblett , J. Hanson , and M. Davis , “Reflecting Reality: Pager Simulations in Undergraduate Education,” Clinical Teacher 11 (2014): 421–424, 10.1111/tct.12185. 25212925

[26] E. I. Emin , E. Emin , A. Bimpis , et al., “Teaching and Assessment of Medical Students During Complex Multifactorial Team‐Based Tasks: The “Virtual On Call” Case Study,” Advances in Medical Education and Practice 13 (2022): 457–465, 10.2147/AMEP.S357514. 35547870 PMC9084906

[27] J. Fisher , R. Martin , and D. Tate , “Hands On + Hands Free: Simulated On‐Call Interaction,” Clinical Teacher 11 (2014): 425–428, 10.1111/tct.12180. 25212926

[28] P. Hamilton , C. Coey‐Niebel , J. McCaig , et al., “Evaluation of Inter‐Professional Education (IPE) With Medical, Nursing and Pharmacy Students Through a Simulated IPL Educational Intervention,” International Journal of Clinical Practice 75, no. 11 (2021): e14725, 10.1111/ijcp.14725. 34382304

[29] N. Harrison , S. Sharma , G. Heppenstall‐Harris , et al., “A Virtual 'Hour on‐Call': Creating a Novel Teaching Programme for Final‐Year Undergraduates During the COVID‐19 Pandemic,” Future Healthcare Journal 8 (2021): S2, 10.7861/FHJ.8-1-S2.

[30] J. Manalayil , A. Muston , A. Ball , and D. Chevalier , “1HR On‐Call—Using Simulated On‐Call to Underpin Experiential Learning in Final Year Medical Students,” Journal of European CME 9 (2020): 1832749, 10.1080/21614083.2020.1832749. 33224625 PMC7655053

[31] L. Misquita , L. Millar , and B. Bartholomew , “Simulated On‐Call: Time Well Spent,” Clinical Teacher 17 (2020): 629–637, 10.1111/tct.13148. 32202375

[32] M. C. McGlynn , H. R. Scott , C. Thomson , S. Peacock , and C. Paton , “How We Equip Undergraduates With Prioritisation Skills Using Simulated Teaching Scenarios,” Medical Teacher 34 (2012): 526–529. 22452281 10.3109/0142159X.2012.668235

[33] K. Moqeem , D. Ricardo , and M. Bradley , “What's a Bleep?: Introduction of Twilight Shadowing Shifts in the Medical School Curriculum,” Future Healthcare Journal 10, no. Supplement 3 (2023): S69. 10.7861/fhj.10-3-s69 PMC10884713 38406714

[34] M. M. Nichols , A. Radcliffe , and A. Daniel , “Virtual On‐Call: Use of Low‐Fidelity Simulation to Improve Preparedness for Practice,” Cureus 16 (2024): e73916. 39697946 10.7759/cureus.73916 PMC11655096

[35] R. Nitiahpapand , T. Western , M. Byrne , and L. Curran , “Virtual On‐Call Increases Medical Student Preparedness for Practice,” Medical Education 54 (2020): 482, 10.1111/medu.14099. 32167187

[36] N. Ramsden , J. Newman , R. Cooper , and A. Wilson , “‘An Hour on Call’—Simulated Medical Education,” Future Hospital Journal 3 (2016): s41, 10.7861/futurehosp.3-2s-s41. 31098270 PMC6465916

[37] J. Seale , S. C. Ragbourne , N. Purkiss Bejarano , et al., “Training Final Year Medical Students in Telephone Communication and Prioritization Skills: An Evaluation in the Simulated Environment,” Medical Teacher 41, no. 9 (2019): 1023–1028, 10.1080/0142159X.2019.1610559. 31124719

[38] R. Scott , S. D. Chumbley , M. Miles , C. Beattie , and A. Grewal , “On‐Call Simulation: Evaluating Cost and Impact,” Clinical Teacher 21 (2024): e13807, 10.1111/tct.13807. 39255975

[39] B. P. Tano and J. Laraman , “Virtual On‐Call: Does a Simulated On‐Call Session Increase the Preparedness of Final‐Year Medical Students?,” Future Healthcare Journal 9 (2022): S46, 10.7861/fhj.9-2-s46. PMC9601071 36310989

[40] V. Langendyk , “Not Knowing That They Do Not Know: Self‐Assessment Accuracy of Third‐Year Medical Students,” Medical Education 40 (2006): 173–179, 10.1111/j.1365-2929.2005.02372.x. 16451246

[41] R. A. Denisco , J. N. Drummond , and J. S. Gravenstein , “The Effect of Fatigue on the Performance of a Simulated Anesthetic Monitoring Task,” Journal of Clinical Monitoring 3 (1987): 22–24, 10.1007/bf00770879. 3819792

[42] R. Smith‐Coggins , M. R. Rosekind , S. Hurd , and K. R. Buccino , “Relationship of Day Versus Night Sleep to Physician Performance and Mood,” Annals of Emergency Medicine 24 (1994): 928–934, 10.1016/s0196-0644(94)70209-8. 7978567

[43] M. Maier , L. Lawrie , D. Powell , P. Murchie , and J. L. Allan , “Lengthy Shifts and Decision Fatigue in Out‐of‐Hours Primary Care: A Qualitative Study,” Journal of Evaluation in Clinical Practice 31 (2025): e70050, 10.1111/jep.70050. 40078025 PMC11904385

[44] D. Synnott , S. Cavallari , K. Synnott , and N. Coakley , “Stepping Into the Night: The Preparedness of Newly Qualified Doctors for Out‐of‐Hours Work,” Clinical Teacher 22 (2025): e70035, 10.1111/tct.70035. 39933552

[45] A. Baten , C. Bleeker‐Rovers , F. van den Heijkant , J. de Graaf , and C. R. Fluit , “Residents' Readiness for Out‐of‐Hours Service: A Dutch National Survey,” Netherlands Journal of Medicine 76 (2018): 78–83. 29515005

