# Supplementary material for: Educational Interventions to Prepare Undergraduate Students for Out‐of‐Hours Practice as a Newly Qualified Doctor in the United Kingdom: A Scoping Review
Source: Clin Teach. 2025 Dec 17;23(1):e70299. doi: 10.1111/tct.70299 (PMC12710567; doi:10.1111/tct.70299)
Supplement: Supplementary file 1 — Appendix S1: Search Strategy for Medline [file TCT-23-e70299-s002.docx]

# **Appendix 1 – Search Strategy for Medline**

| **1** | **medical education/** |
| --- | --- |
| **2** | **medical student/** |
| **3** | **medical school/** |
| **4** | **medical education.tw.** |
| **5** | **medical student*.tw.** |
| **6** | **(undergraduate adj2 medic*).tw.** |
| **7** | **medical degree*.tw.** |
| **8** | **(student* adj2 (doctor* or physician*)).tw.** |
| **9** | **junior doctor*.tw.** |
| **10** | **(foundation adj3 doctor*).tw.** |
| **11** | **((recently qualified or newly qualified) adj3 doctor*).tw.** |
| **12** | **medical graduate*.tw.** |
| **13** | **(medical trainee* or trainee doctor*).tw.** |
| **14** | **((F1 or F2 or FY1 or FY2) adj3 doctor*).tw.** |
| **15** | **or/1-14** |
| **16** | **out-of-hours.tw.** |
| **17** | **out-of-office hours.tw.** |
| **18** | **out-of-normal hours.tw.** |
| **19** | **out-of-normal working hours.tw.** |
| **20** | **after hours.tw.** |
| **21** | **(work* adj2 weekend*).tw.** |
| **22** | **(work* adj2 night*).tw.** |
| **23** | **nightshift.tw.** |
| **24** | **(shift adj2 (work* or rotation or pattern*)).tw.** |
| **25** | **night shift*.tw.** |
| **26** | **twilight.tw.** |
| **27** | **long day*.tw.** |
| **28** | **handover*.tw.** |
| **29** | **ward cover.tw.** |
| **30** | **bleep.tw.** |
| **31** | **on-call.tw.** |
| **32** | **or/16-31** |
| **33** | **15 and 32** |
| **34** | **limit 33 to yr="2009 -Current"** |
| **35** | **United Kingdom/** |
| **36** | **Great Britain/** |
| **37** | **Ireland/** |
| **38** | **Northern Ireland/** |
| **39** | **(national health service* or NHS*).ab,ad,in,ti.** |
| **40** | **(gb or "g.b." or britain* or (british* not "british columbia")).ab,ad,in,ti.** |
| **41** | **(UK or "U.K." or United Kingdom*).ab,ad,in,ti.** |
| **42** | **(England* not "new England").ab,ad,in,ti.** |
| **43** | **(Ireland or Irish or Scotland or Scottish or ((Wales or "South Wales") not "new South Wales") or Welsh).ab,ad,in,ti.** |
| **44** | **(bath or "bath's" or ((birmingham not alabama*) or ("birmingham's" not alabama*) or bradford or "bradford's" or brighton or "brighton's" or bristol or "bristol's" or carlisle* or "carlisle's" or (cambridge not (massachusetts* or boston* or harvard*)) or ("cambridge's" not (massachusetts* or boston* or harvard*)) or (canterbury not zealand*) or ("canterbury's" not zealand*) or chelmsford or "chelmsford's" or chester or "chester's" or chichester or "chichester's" or coventry or "coventry's" or derby or "derby's" or (durham not (carolina* or nc)) or ("durham's" not (carolina* or nc)) or ely or "ely's" or exeter or "exeter's" or gloucester or "gloucester's" or hereford or "hereford's" or hull or "hull's" or lancaster or "lancaster's" or leeds* or leicester or "leicester's" or (lincoln not nebraska*) or ("lincoln's" not nebraska*) or (liverpool not (new south wales* or nsw)) or ("liverpool's" not (new south wales* or nsw)) or ((london not (ontario* or ont or toronto*)) or ("london's" not (ontario* or ont or toronto*)) or manchester or "manchester's" or (newcastle not (new south wales* or nsw)) or ("newcastle's" not (new south wales* or nsw)) or norwich or "norwich's" or nottingham or "nottingham's" or oxford or "oxford's" or peterborough or "peterborough's" or plymouth or "plymouth's" or portsmouth or "portsmouth's" or preston or "preston's" or ripon or "ripon's" or salford or "salford's" or salisbury or "salisbury's" or sheffield or "sheffield's" or southampton or "southampton's" or st albans or stoke or "stoke's" or sunderland or "sunderland's" or truro or "truro's" or wakefield or "wakefield's" or wells or westminster or "westminster's" or winchester or "winchester's" or wolverhampton or "wolverhampton's" or (worcester not (massachusetts* or boston* or harvard*)) or ("worcester's" not (massachusetts* or boston* or harvard*)) or (york not ("new york*" or ny or ontario* or ont or toronto*)) or ("york's" not ("new york*" or ny or ontario* or ont or toronto*))))).ab,ad,in,ti.** |
| **45** | **(bangor or "bangor's" or cardiff or "cardiff's" or newport or "newport's" or st asaph or "st asaph's" or st davids or swansea or "swansea's").ab,ad,in,ti.** |
| **46** | **(aberdeen or "aberdeen's" or dundee or "dundee's" or edinburgh or "edinburgh's" or glasgow or "glasgow's" or inverness or (perth not australia*) or ("perth's" not australia*) or stirling or "stirling's").ab,ad,in,ti.** |
| **47** | **(armagh or "armagh's" or belfast or "belfast's" or lisburn or "lisburn's" or londonderry or "londonderry's" or derry or "derry's" or newry or "newry's").ab,ad,in,ti.** |
| **48** | **35 or 36 or 37 or 38 or 39 or 40 or 41 or 42 or 43 or 44 or 45 or 46 or 47** |
| **49** | **(exp "arctic and antarctic"/ or exp oceanic regions/ or exp western hemisphere/ or exp africa/ or exp asia/ or exp "australia and new zealand"/) not (united kingdom/ or europe/)** |
| **50** | **48 not 49** |
| **51** | **34 and 50** |
