# Supplementary material for: Educational Interventions to Prepare Undergraduate Students for Out‐of‐Hours Practice as a Newly Qualified Doctor in the United Kingdom: A Scoping Review
Source: Clin Teach. 2025 Dec 17;23(1):e70299. doi: 10.1111/tct.70299 (PMC12710567; doi:10.1111/tct.70299)
Supplement: Supplementary file 2 — Appendix S2: Medical School Annual Return Responses 2017/2018 [file TCT-23-e70299-s001.docx]

**Appendix 2. Medical School Annual Return Responses 2017/18.**

| **Medical School** | **Action/ Agreed Action/ Recommendation** |
| --- | --- |
| Aberdeen | We already provide early curricular experience for out-of-hours working through shadowing of Hospital at Night teams. It is our expectation and requirement that students undertaking an assistantship should routinely gain experience of out-of-hours working |
| Barts | We have carried out extensive consultation with our students and NHS academic/clinical staff before approving a policy to cover Out-of-hours attendance through the MBBS course. In years one and two there is very little Out-of-hours activity. In year three it is suggested that students undertake three late night or weekend (one day only) attachments to observe unselected medical (x2) and surgical (x1) take. In year four, students experience four late night or weekend (one day only) attachments. For example, it is expected that students will need to attend out-of-hours on at least one occasion during Obstetrics and Gynaecology placement to follow progression of labour. In year five, there is more extensive Out-of-hours work in the lead up to FY1 year. A minimum of one Out-of-hours’ session per month of the taught course is suggested. |
| Birmingham | In Year 3, students have always been involved in evening on-call until 10pm. However, **participating in out-of-hours working had started to become less of a routine part** of the culture of undergraduate education in our NHS Academies. To some extent this may be **due to changes in the way out-of-hours working is managed within the Trusts**. This has led to anecdotal concern in some Academies that having students participate in out-of-hours working may have a detrimental effect on busy medical staff.  In order to develop out-of-hours working experience further however, we have introduced one “12 hour weekend on call shift” to the third year medical students  in their first hospital placement as a pilot in the current academic year. The majority of the hospital Trusts were enthusiastic about the new initiative and we will be reviewing feedback from both the students and the Academies.  Within year 4 it has always been possible for, and we have encouraged, students to gain experience in out-of-hours working with specialty teams although this has not been a compulsory part of the placements. However, we plan to make it so subject to the success of the pilot in year 3, when we review our 4th year curriculum.  In Year 5 and the assistantship, students have to do a minimum of 1 night on- call and 1 second-on shift. This is monitored to ensure students do so. |
| Bristol | Our Year 5 students have undertaken student assistantships of 4-5 weeks duration in both medicine and surgery since the 2010-11 academic year. Within each assistantship students are attached to a specific foundation doctor within teams and often effectively take the place of the foundation doctor if (s)he is on leave. These assistantships have always included time specifically in out-of-hours working (including night cover) by following individual foundation doctors on-call patterns. These assistantships are firmly embedded in local academy delivery of undergraduate teaching, are enjoyed by students and are rewarding to deliver for teachers. |
| Buckingham | Our *‘Standards for Block Timetables’* define the experiences that students must have to meet the outcomes defined by the GMC, including the required time input from clinical teachers.  They include requirements for out-of-hours experience, and experience relating to patient journeys. This occurs widely across the course in Phase 2, and students have programmed out- of-hour experience in most blocks. This is most prominent in the ‘Acute Care’ block which is designed around a shift system, and in the plans for student assistantship, which will also be shift-based. |
| Cambridge | The school has developed a 6 week apprenticeship block at the end of the final clinical year to allow these areas to be specifically addressed. All of our students obtain out-of-hours experience when on placement in regional trusts, through joining rotas and working on night shifts (for example, during their Year 4 and Year 6 emergency care and acute surgery experiences). |
| Cardiff | All students are encouraged to participate in ‘out-of-hours’ working, from year 3, but the expectation is increased as the students’ progress from year to year. Students are encouraged to do this especially in Paediatrics and Obstetrics and Gynaecology (Year 4) and then in Year 5 Student Assistantship modules when students spend time in Emergency Units and Medical and Surgical Emergency  Assessment units, working with clinical teams on a full time basis. |
| Edinburgh | Out of ours working has had a limited impact as we had already timetabled our senior students to experience a full variety of shift types, including evenings, weekends and Hospital at Night (HAN) working. We are working to increase this type of experience and enhance the consistency, quality and equality of exposure. |
| Exeter | In year 5 we expect the students to do one OOH shift per 6-week block. In secondary care this has been well received by the students. They report feeling well-supported and gaining huge amounts of experience from working in the hospital in the evenings and at night. |
| Glasgow | Our Preparation for Practice block has become a staple part of our course (post- finals in Year 5) and will include a requirement to get experience of out-of-hours work – both ward cover and on-take. |
| Hull York | Out-of-hours care is built into medical student clinical placements already, and students have consistently given positive feedback on this experience. |
| Imperial | The School already strongly encourages students to avail themselves of the many opportunities that exist for out-of-hours training, though this has to be carefully balanced with student health, safety and wellbeing and the many other commitments that they have outside of their studies. The School is in the process of producing guidance for placement leads on this topic. |
| Keele | we have mandatory out-of-hours experience during Year 5 assistantships. |
| King’s College London | Not specified |
| Lancaster | Out-of-hours work remains an area under development but we have developed placements within hospital outside of core hours. |
| Leeds | Working out-of-hours shifts are also a mandatory requirement for all final year placements, providing a complementary clinical experience to the routine daytime patient review and clinical tasks. This is particularly prominent in some specialty placements (e.g. Emergency Medicine, obstetrics). |
| Leicester | The curriculum of our assistantships is based on the current Foundation Programme. In particular, our students will shadow FY1/2 doctors in challenging environments and work to varying rotas including out-of-hours **[R5.3h]**. |
| Liverpool | There are multiple opportunities for out-of-hours working for students within the current curriculum; all placement sites are encouraged to offer out-of-hours shifts where feasible. These are compulsory for placements undertaken at the Women’s Hospital (undertaken in Year 3) which includes one night and one weekend shift, and as part of many Year 5 placements.  The ward shadow and acute blocks of fifth year both incorporate shadowing out-of-hours and on call shifts under the direct supervision of qualified doctors |
| Manchester | This is hospital based and very well evaluated but we would like to expand this into General Practice. The challenge is to create a community equivalent, particularly with Out-of-hours (OOH) provision. OOH providers use ad-hoc clinicians for sessions and it is very challenging to set up long-term supervisory relationships OOH for a prolonged period. |
| Newcastle | During this it is a requirement for the students to gain experience of working ‘out-of-hours’. Within the HBP study guide there is a section (see below) giving guidance about the benefits of this and what is expected of the students. We wondered whether the GMC may consider this as an area of good practice  “At some point through the course you will be required to be part of a team responsible for unselected admissions and for covering patients out-of-hours. You will be supervised throughout this period by the medical team responsible for the patients. “ |
| Oxford | After our final examinations in medicine and surgery all students carry out a student assistantship where they work alongside a foundation doctor in our main NHS partner trust. Students are required to record activities which result in them meeting graduate learning outcomes and to ‘ticket’ their supervising foundation  doctor. The supervisor completes a short report form and in return the medical school reports on their participation in teaching/supporting the learning of medical students. |
| Plymouth | Ensure all final year students undertake 3 out-of-hours placements in secondary care |
| Plymouth | The recent GMC visit explored the issue of out-of-hours clinical experience. We had previously undertaken these on a ‘recommended’ basis for students. Since these constructive discussions, we have put forward a proposal to the medical programmes committee that these become a mandatory requirement in Year 5 to ensure that students have direct experience of this important aspect of junior doctor practice. This is in line with the GMC team’s recommendation. |
| Queen’s Belfast | During clinical attachments from years 3-5 students are encouraged to attend hospital units out-of-hours |
| Sheffield | We are currently in transition from short to long placements. The long placements will help provide more opportunities for H@N and out-of-hours which are currently undertaken from Y3-4 to the final Student Assistantship where students are shadowing the F1 of their future job. Out-of-hours is a requirement of the acute and critical care block as well Emergency Medicine. |
| Southampton | Students are required to undertake out-of-hours experiences in both the Assistantship module and the new Year 4 Acute Care module. While it is not compulsory for students to undertake out-of-hours experiences in other modules, it is encouraged, and many students report excellent learning experiences from having attended their placements out of day time working hours. |
| St Andrews | Exclude |
| St George’s | There is no specific challenge or impact of the new requirement regard out-of- hours work experience since the SGUL curriculum has long required students to gain this, specifically in final year assistantships |
| Swansea | **Swansea GEM curriculum offers 3 Junior Assistant ship, each lasting 5 weeks and a 5-week Senior assistantship before their placement as a F1 doctor. They are encouraged to attend out-of-hours with the team that they are working with. Mandatory out-of-hours work is build in the speciality attachments and LOCS provides similar opportunities in early years** |
| University College London | Not specified; We are using the recommendations from the London Medicine and Healthcare report “Providing effective undergraduate medical clinical placements in London” |
| University of East Anglia | For the out-of-hours in hospital placement we have had a policy to expand our utilisation of out-of-hours experience in many different disciplines throughout the 5 years of study. For example every student currently will have delivery suite experience during obstetrics that are evenings or weekends. Other examples include neurology, chest and respiratory medicine attachments (where students are offered post take ward round slots that start at 5pm and run through the evenings), and during acute medicine where students are offered to attend at least one night and one weekend slot. During their student assistantship module, they are offered experience shadowing ‘hospital at night’ shifts. Out-of-hours shifts are not currently a compulsory component (for example if a student has reasonable adjustments) however we are consulting the student body about making a limited number compulsory out-of-hours sessions in each academic year in the future |
| Warwick | Students are timetabled out-of-hours sessions in acute medicine (phase 3) and obstetrics (phase 2 and 3). Further experience is encouraged in phase 3 but not required. The main challenge here is the nature of our graduate entry students  means that many of them work part-time jobs alongside their studies in evenings and at weekends. Other students have childcare and caring responsibilities. This is usually successfully managed with sufficient notice of timetabled sessions and with individual staff input to deal with challenges for specific individuals. |
